# Endosulfan exposure alters transcription of genes involved in the detoxification and stress responses in *Physella acuta*

**DOI:** 10.1038/s41598-020-64554-8

**Published:** 2020-05-12

**Authors:** María Alonso-Trujillo, Ana-Belén Muñiz-González, José-Luis Martínez-Guitarte

**Affiliations:** Grupo de Biología y Toxicología Ambiental. Facultad de Ciencias. Universidad Nacional de Educación a Distancia, UNED. Senda del Rey 9, 28040 Madrid, Spain

**Keywords:** Cell biology, Molecular biology, Environmental sciences

## Abstract

Endosulfan is a persistent pesticide that has been in use for more than five decades. During this time, it has contaminated soil, air, and water reservoirs worldwide. It is extremely toxic and harmful to beneficial non-target invertebrates, aquatic life, and even humans upon consumption, which is one of the many dangers of this pesticide since it biomagnifies in the food chain. The effects of three endosulfan concentrations (1, 10, and 100 µg/L) on the freshwater snail *Physella acuta*, an invasive cosmopolitan species, were examined over a week-long exposure period. Alterations in the expression of ten genes related to stress and xenobiotic detoxification were measured against the endogenous controls *rpL10* and *GAPDH* by Real-Time polymerase chain reaction. Four genes are described here for the first time in this species, namely *Hsp60*, *Grp78*, *GSTk1*, and *GSTm1*. The rest of genes were *Hsp90*, *sHsp16.6*, *cyp2u1*, *cyp3a7*, *cyp4f22*, and *MRP1*. *cyp2u1*, *sHsp16.6*, and *Grp78* expression were all altered by endosulfan. These results suggest a low pesticide concentration activates the acute response in *P. acuta* by affecting detoxification and stress responses and alter endoplasmic reticulum function and lipid metabolism. Furthermore, the newly identified genes extend the number of processes and cellular locations that can be analyzed in this organism.

## Introduction

Endosulfan, also known as Thiodan or Thionex, is a polychlorinated compound used as a pesticide or acaricide. It has been in use since the 1950s in China, the European Union, Australia, Mexico, the United States, and India^1^ for crops including maize, soybeans, tomatoes, and cotton^2^. It is particularly effective in removing aphids, fruit worms, beetles, leafhoppers, moth larvae, and whiteflies^3^. However, its highly toxic properties can potentially harm humans and wildlife^4,5^. Further, due to its semi-volatility, endosulfan is often found thousands of kilometers away from its intended area of action in soil, water reservoirs, sediments, and even Arctic and Antarctic lakes. These findings prove its persistent character; indeed, it is listed as a persistent organic pollutant in the atmosphere, soil, and water by the Stockholm Convention^6^. For this reason, it has been banned in over 80 nations. However, one of the main problems with pesticides as endosulfan is the fact that they are persistent and can be dispersed from air to water bodies. The environmental presence of endosulfan has been evaluated by Mohamed *et al*., 2019^7^ showing values from 0.036 μg/L to 62.3 μg/L in water bodies. Indeed, it is an extremely toxic and harmful compound to beneficial non-target invertebrates living in aquatic media^8–10^.

Endosulfan is commercially available as a mixture of its isomers, α and β, but higher environmental concentrations correspond to α-endosulfan, since β-endosulfan is naturally converted into α-endosulfan in a slow way due to its higher stability^3^. Although they share similar chemical and physical properties, under aerobic conditions the β-endosulfan half-life is approximately 28 days, whereas α-endosulfan lasts up to 157 days^11^. Certain organisms or chemicals can metabolize endosulfan through oxidation to endosulfan sulfate, which is more toxic and stable^6^. Endosulfan is very toxic to aquatic organisms, especially fish and invertebrates^12–14^. It irreversibly affects fish gills, hindering oxygen intake^15^, and interferes with the endocrine system^16^. It is also neurotoxic, since it affects mouse brain development^17^. Although the effects of endosulfan have been extensively analyzed in several species, knowledge about how it affects certain animal groups remains modest.

Mollusks are common model organisms in toxicology, but usually bivalves such as the blue mussel (*Mytilus edulis*) or the Manila clam (*Ruditapes philippinarum*) are used to study pollution in seawater. On the other hand, gastropods like the great pond snail (*Lymnaea stagnalis*) can provide toxicity information related to freshwater reservoirs^18,19^. The tadpole snail *Physella acuta* (Draparnaud, 1805), also known as *Physa acuta*, is a cosmopolitan freshwater species present in lakes and ponds worldwide. It is a hermaphroditic panpulmonate hygrophilid snail that belongs to the Physidae family. Due to its resilience, its ability to be maintained under laboratory conditions, and its differential sensitivity to respond to toxicants, it could be a good model organism for aquatic toxicity testing as we have shown previously^20–23^. Therefore, we selected this species to analyze the impact of endosulfan on messenger RNA (mRNA) levels in gastropods.

Detoxification mechanisms remove toxic substances from the cell in a three-phase process. Phase I involves oxidation, reduction, and/or hydrolysis^24^, all of which render a more hydrophilic compound. The main phase I enzymes are part of the cytochrome P450 superfamily^25^, highly conserved monooxygenases that contain a heme prosthetic moiety^26^. We assayed three members of this family in this study: cytochrome P450 2u1 (*cyp2u1*), cytochrome P450 3a7 (*cyp3a7*), and cytochrome P450 4f22 (*cyp4f22*), all of which were previously identified in our laboratory^22^. During phase II, conjugation to glutathione, acetylation, sulfation, or glucuronidation either neutralizes or generates chemicals that can be transported out of the cell^27^. Myriad enzymes play a role in phase II, such as sulfotransferases, methyltransferases, and acetyltransferases^27^, but the best known are the glutathione-S-transferases (GSTs). They aid in the degradation or excretion of certain toxic substances^28^. Cytosolic GSTs are divided into thirteen classes based upon their structure (alpha, beta, delta, epsilon, zeta, theta, mu, nu, pi, sigma, tau, phi, and omega); there is also one mitochondrial class (kappa^29^). Similar to cytochrome P450s, GSTs are involved in diverse cellular processes depending on the substrate^30^, and so they are not restricted to detoxification. In this study, we identified two genes that code for novel GSTs in this species, *GSTm1* and *GSTk1*, and evaluated their response to endosulfan. Finally, in phase III unwanted molecules are transported outside the cell by ATP-binding cassette transporters, which transport substances that are bound to glutathione, glucuronide, or sulfur^31^; there is a subgroup known as multidrug resistance proteins because there are involved in resistance to different drugs^32^. *MRP1* expression, also referred to as ABCC1, participates in detoxification and has been described in numerous invertebrates, including *P. acuta*^22,33^. We selected it as a phase III representative.

When exposed to non-favorable or stressful conditions, cells must cope with the adverse conditions by utilizing various proteins, including heat shock proteins (Hsps). This diverse and highly conserved group reacts to environmental changes and performs different functions to protect cells from stress. For instance, they act as chaperones and assist with folding or unfolding proteins as well as in the assembly of protein complexes, and they are also involved in cell cycle signaling^34,35^. Hsps are categorized into five families according to their molecular weight: Hsp100s, Hsp90s, Hsp70s, Hsp60s, and small heat shock proteins (sHsps^36^). There are also other Hsps, such as Hsp40 and Hsp10, which act as cochaperones for other Hsps. Here we analyzed *Hsp60*, *Grp78*, *Hsp90*, and *sHsp16.6*, which code Hsp60, Hsp70, Hsp90, and sHsp family proteins, respectively. Hsp60 is a mitochondrial chaperone involved in folding and preserving correct cellular protein conformations^37^. Glucose-regulated protein 78k Da (Grp78), also known as BiP, is an endoplasmic reticulum chaperone related to the Hsp70 family that ensures the quality of proteins leaving that organelle and activates transmembrane signaling pathways that maintain homeostasis under stress^38^. *Hsp90* codes for a protein that belongs to the Hsp90 family. In invertebrates, it aids in the maturation, folding, and maintenance of molecules as well as in stabilizing hormonal receptors^39^ that affect metabolic processes. sHsp16.6 exhibits a characteristic sHsp α-crystallin domain^40^. These proteins do not require ATP but rather cooperate with other ATP-dependent chaperones, such as Hsp60 or Hsp10, to remove protein aggregates and regenerate their correct shape to restore their activity.

This study aimed to determine, at the cellular level, the ability of the mollusk to manage the presence of the organochlorine pesticide endosulfan. By using Real-Time polymerase chain reaction (RT-PCR), we analyzed the mRNA levels of ten genes involved in detoxification and stress in adult *P. acuta* exposed to non-lethal endosulfan concentrations (1, 10, or 100 μg/L) for 7 days. The analyzed genes code proteins involved in the three detoxification phases and the four Hsp families to establish whether endosulfan altered their transcript levels. Further, we examined genes for proteins from the cytosol, endoplasmic reticulum, and mitochondria to evaluate their potential as putative molecular biomarkers of damage to these cell compartments or organelles. This type of study is essential because of the role of *P. acuta* in aquatic ecosystem food webs, endosulfan persistence, and the poor knowledge about its effects on mollusks. Determining how it affects *P. acuta* could help show new mode of actions in other invertebrates.

## Results

### Identification of new genes involved in detoxification and stress responses

**Figure 1:**
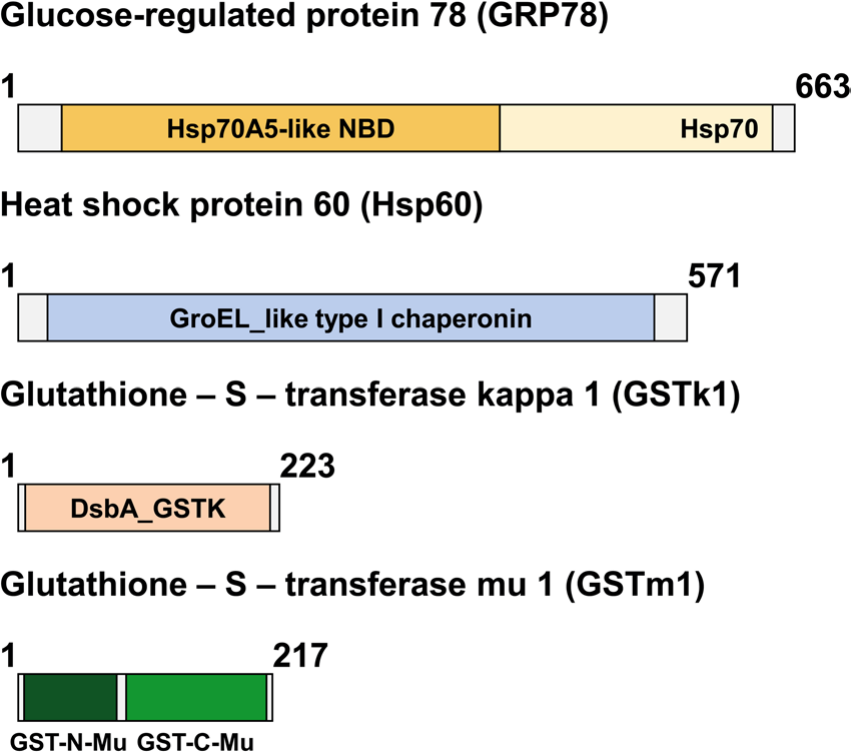
Structure and conserved domains of the identified *P. acuta* proteins in this study. A scheme of the protein from each open reading frame is shown with the different motifs that characterize them. The domains are defined by the CCD functional classification of proteins.

From analysis of the *P. acuta* transcriptome, we identified four sequences of interest related to detoxification and stress responses. A comparison of the assembled sequences to GenBank database using Blast2GO program^41^ was carried out. The ORFs of some of the sequences showed similarity to proteins involve in stress response and detoxification mechanisms so they were selected as first candidates to be tested. The mRNA and predicted proteins for each sequence are shown in Table 1. Protein sequence comparison using blast demonstrated homology with two GST members (GSTk1 and GSTm1) and two Hsp members (HSP60 and GPR78). Both GSTs were identified by homology to GST kappa and GST mu in other mollusks and the presence of a GST kappa motif and N- and C-terminal GST mu motifs, respectively (Fig. 1). On the other hand, the third sequence was homologous to Hsp60 from *Biomphalaria glabrata* and a GroEL-like type I chaperonin, characteristic of this Hsp. Finally, the last sequence coded for a protein with homology to GRP78 from *Aplysia californica*; it exhibited an HSPA5 motif, which is related with members of the Hsp70 family associated with the endoplasmic reticulum. Considering this data, we concluded that the sequences code for *P. acuta* proteins GSTk1, GSTm1, Hsp60, and GRP78.

**Table 1:** Sequence characteristics and protein homology. GenBank accession numbers are provided.

Acc number	Gene	RNA size (base pairs) /Protein Size (amino acids)	Homologous	Identity/Similarity
MK814856	Glutathione–S–transferase kappa 1 (GSTk1)	1254 bp/223aa	Predicted: Glutathione S-transferase kappa 1-like *Aplysia californica* - XP_005109181	57%/75%
MK814857	Glutathione–S–transferase mu 1 (GSTm1)	1329 bp/217aa	Predicted: Glutathione S-transferase Mu 1-like *Biomphalaria glabrata* - XP_013097111	71%/83%
MK814858	Heat shock protein 60 (Hsp60)	2309 bp/571aa	60 kDa heat shock protein, mitochondrial-like *Biomphalaria glabrata* - NP_001298236	95%/98%
MK814859	Glucose-regulated protein 78 (GRP78)/Binding immunoglobulin protein (BiP)	3024 bp/663aa	Endoplasmic reticulum chaperone BiP precursor *Aplysia califórnica* - NP_001191581	91%/95%

### Endosulfan effects on detoxification genes

**Figure 2:**
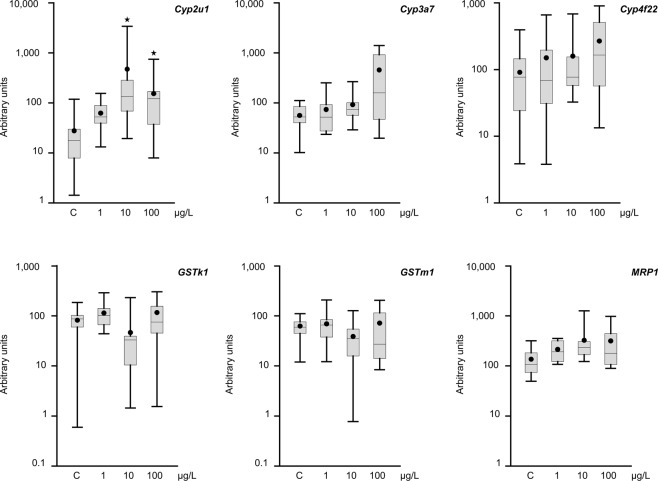
Transcript levels of phase I (*cyp2u1, cyp3a7*, and *cyp4f22*), phase II (*GSTk1* and *GSTm1*), and phase III (*MRP-1*) metabolism genes in *P. acuta* adults after *in vivo* exposure to endosulfan for 7 days at 19 °C. Transcriptional activity was quantified by RT-PCR using *rpL10* and *GAPDH* as reference genes. The comparison was performed with the solvent-exposed controls and among treatments. Whisker boxes are shown. Each box corresponds to nine individuals. The median is indicated by the horizontal line within the box, and the 25th- and 75th-percentiles are indicated by the boundaries of the box. The highest and lowest results are represented by the whiskers. The small circle inside the box denotes the mean. Differences with controls (asterisk) are indicated (p < 0.05).

No mortality was detected to any of the treatments and all the animals continued to display normal activities in the moment of be frozen. Since endosulfan is a pesticide, we analyzed activation of the detoxification mechanism by studying the mRNA levels of phase I (*cyp2u1*, *cyp3a7*, *cyp4f22*), phase II (*GSTk1*, *GSTm1*), and phase III (*MRP1*) metabolism genes. Of these genes, only *cyp2u1* was altered, specifically, with increased expression for the 10 and 100 µg/L concentrations compared to control (Fig. 2). While the remaining genes were not statistically significantly altered, *cyp3a7* and *cyp4f22* showed a trend to increase with endosulfan treatment. These results suggest that even low endosulfan levels can activate phase-I-related genes.

### Endosulfan effects on stress-response genes

**Figure 3:**
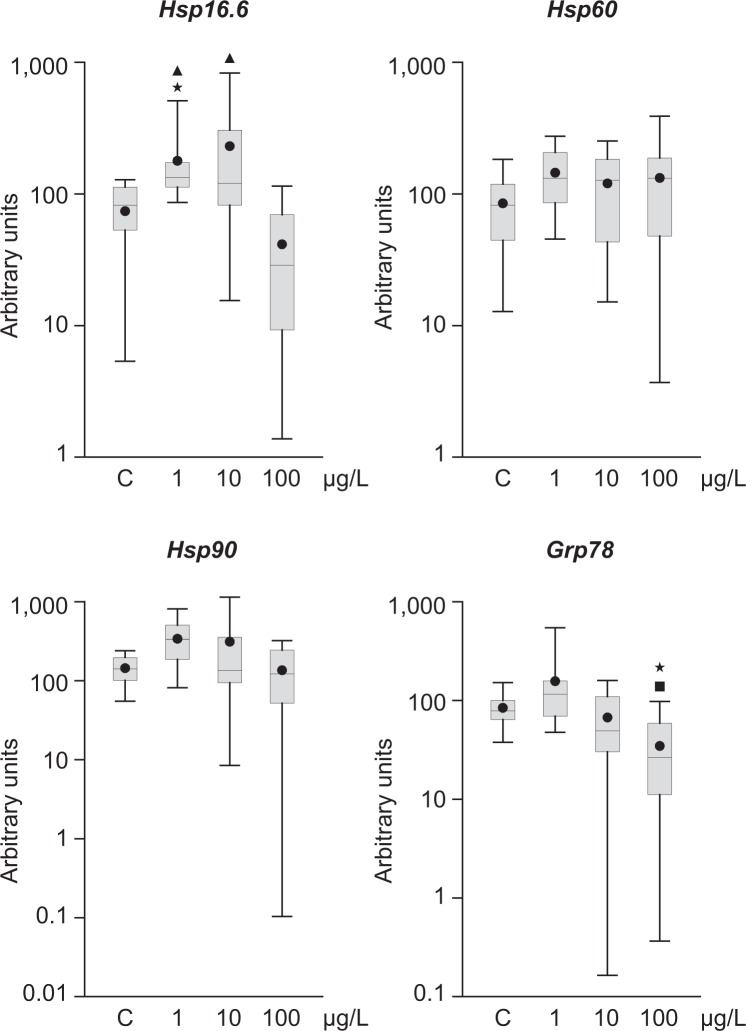
Transcriptional activity of *hsp16.6*, *hsp60*, *hsp90*, and *GRP78* in adult *P. acuta* after *in vivo* exposure to endosulfan at 19 °C for 7 days. Levels of mRNA were normalized using *rpL10* and *GAPDH* as reference genes. The analysis was performed by comparing the values with solvent-exposed controls and among treatments. The n for each box is nine. The median is indicated as a horizontal line within the box while the 25th- and 75th-percentiles are shown by the boundaries. The whiskers represent the highest and lowest results. The small circle indicates the mean. Differences with control (asterisk), 1 μg/L (square), or 100 μg/L (triangle) are indicated (p < 0.05).

The stress response is one of the first systems that protects the cell. To analyze it, we examined the levels of four genes from different Hsp families, the most important proteins involved in stress. The small Hsp gene, *Hsp16.6*, was significantly increased compared to control for 1 µg/L endosulfan (Fig. 3). However, its expression was substantially decreased at 100 µg/L compared to the 1 and 10 µg/L concentrations but not control. *Grp78* was significantly decreased for 100 µg/L endosulfan compared to control and the 1 µg/L concentration (Fig. 3). Endosulfan treatment did not affect *Hsp60* or *Hsp90* expression.

## Discussion

Endosulfan is a toxicant that has long been known to damage wildlife. As a pesticide, it alters acetylcholinesterase activity in insects, but there is poor knowledge of its mode of action in other invertebrates at the cellular level. In this study, we analyzed the cellular response of the snail *P. acuta* and observed that stress response and detoxification mechanisms are activated during short-term exposure to low endosulfan levels. Furthermore, we identified four novel genes that can be useful for toxicity evaluation. Two of these genes code for GST enzymes, while the other two code for Hsps. GSTk1, also known as mitochondrial GST, appears in all eukaryotes and is associated with mitochondria and peroxisomes^42^. It is also related to energy and lipid metabolism in *Caenorhabditis elegans*^43^. GSTm1 was intensively studied in humans and mice in relation to toxicity, and the data show that polymorphisms influence toxicant susceptibility^44–46^. However, there is a lack of data on invertebrates, since few published reports analyzed this gene^47^. The other two novel genes belong to the Hsp60 and Hsp70 families. Hsp60 is a protein involved in the stress response in the mitochondria; one study describes its interaction with Hsp10 in other mollusks^48^. GRP78 is a chaperone that belongs to the Hsp70 family; it participates in the unfolded protein response (UBR) in the endoplasmic reticulum and forces the unfolded proteins to refold or degrade using cellular degradation mechanisms^38^. Identification of these genes extends the number of putative biomarkers in *P. acuta* and opens the possibility of analyzing toxicity by detecting mitochondrial damage (Hsp60 and GSTk1), energy and lipid metabolism alterations (GSTk1), phase II detoxification (GSTm1), and stress in the ER (GRP78).

Endosulfan is a neurotoxic insecticide that is harmful to humans^49^, so it is expected that some effects could be observed in other organisms. With the aim of shedding light on endosulfan effects on freshwater mollusks, we analyzed two of the primary toxicant responses. Toxicants usually trigger mechanisms to remove them from the cell, including phase I and phase II detoxification enzymes^27,50^. We studied three phase I Cyp450 genes, and only one of them, *cyp2u1*, was changed (increased) at higher endosulfan concentrations. There was a trend for increased *cyp3a7* expression, but it was not statistically significant. However, it was recently explicated that not only the mean should be considered in the evaluation of different parameters in toxicity^51^, because experimental data show that phenotypic plasticity may change in environmental responses and the changes of variability can be significant, even when the means do not differ, so future research could assist in understanding the importance of these trends. The other detoxification genes, *GSTk1*, *GSTm1*, and *MRP1*, were not altered. Cyp2u1 is associated with the metabolism of long-chain fatty acids, mainly arachidonic acid (AA) and docosahexaenoic acid (DHA^52^), but its exact cellular function is unclear (although it is altered in different diseases^53^). This result suggests that endosulfan modulates lipid metabolism. A recent study reported that the different endosulfan forms alter serum sex steroid hormone levels and the expression of steroidogenesis genes in mice; these changes affect energy metabolism and oxidative stress^54^. However, two other genes analyzed here that are also related to lipid metabolism (*cyp4f22* and *GSTk1*) were not modified. Although Cyp4f22 breaks down fatty acids with a carbon chain longer than 28 carbons^55^, it was not altered at the transcript level. Similarly, since *GSTk1* mRNA was not altered, endosulfan may not affect the processes in which those genes participate. Further research is needed to clarify the meaning of the *cyp2u1* alteration, but it is specific since no alteration was observed by Vinclozolin, a fungicide^22^. On the other hand, low endosulfan levels were relatively tolerable to adult *P. acuta* for this short-medium term exposure because the detoxification-related genes were not induced except for one likely related to lipid metabolism. These results are similar to that observed for Vinclozolin^22^, with no induction of the detoxification genes. It is important to note that the exposure was to low concentrations (ppb range) but the time was shorter. In any case, *Physella acuta* seems to have some resistance to environmental concentrations of pesticides and it is able to manage them without trigger the detoxification response.

The stress response was analyzed by using genes from four Hsp families. We observed changes in *sHsp16.6* and *GPR78* expression. For the sHSP (*sHsp16.6*), the lowest endosulfan concentration increased its expression compared to control, but it was downregulated at the highest concentration compared to the other two. This result could reflect a dynamic where there is an initial transcript increase (induced at 1 µg/L) followed by a later return to lower or perhaps even downregulated levels (observed at 100 µg/L). Endosulfan could temporally induce *sHsp16.6* when the cell encounters a certain concentration, followed by later recovery. Endosulfan induces sHSPs (e.g., *HSP27*) in *Drosophila* and human cells^56,57^. However, there is also a report of *HSP27* downregulation in human cells^58^. It is not possible to establish whether *sHsp16.6* is the same as the previously studied sHSPs because this family has several members named according to molecular weight. Thus, establishing equivalence between different species is complicated. However, *sHsp16.6* did not change with Vinclozolin although a non-statistical significant trend to downregulation was detected^22^. Two additional sHPSs genes were analyzed in this work, with no change for sHSP17 gene and downregulation for *sHSP20.4*. Further research in the expression of sHSP genes will help to elucidate the putative effects of Endosulfan in this family of proteins. In any case, sHSPs could be useful biomarkers when equivalence between species would be defined. The other altered stress gene was *GRP78*, which was downregulated at the highest endosulfan concentration. A previous report in human cultured cells indicated that GPR78 apparently responds differently (i.e., protein induction or no change) to endosulfan depending on the cell type^58^. Here we exposed the animals for longer times, and so the downregulation could be associated with a long-term response. GRP78 is involved in the endoplasmic reticulum unfolded protein response, so this result could reflect some problem in the protein folding in the endoplasmic reticulum. None of the endosulfan concentrations affected the other two Hsp genes, *Hsp60* and *Hsp90*. In *Penaeus monodon*, a shrimp, Hsp90 increased after four-day exposure to low endosulfan concentrations (similar to this study^59^). Thus, the lack of response observed in *P. acuta* could also be due to different physiology or recovery when homeostasis was achieved. Shorter treatment times could help to elucidate which situation occurs although previous studies with Vinclozolin at 24 h rendered no change for this gene^22^. Finally, endosulfan induced Hsp60 in *Lucilia cuprina* and *Musca domestica*^60,61^. Similar to Hsp90, it is possible that different physiology can explain these results, but additional research is needed at shorter times to determine whether early activation could occur.

*P. acuta* is sensitive to low endosulfan levels, which activate a cellular response to manage the compound and prevent chemical stress. The data suggest putative damage in the endoplasmic reticulum folding mechanism and lipid metabolism, but these changes could be related to other cell processes given the multifold roles that the altered genes can perform in the cell. The acute response mechanisms, those activated in a very-short time for an immediate response to the homeostasis alterations due to environmental changes, were adequate to prevent mortality after 7-day exposure, even at the highest concentration. The identification of new putative biomarkers extends the potential pathways that may be analyzed in this species and can help elucidate new endosulfan modes of action. Our data provide novel insights into the ability of freshwater gastropods to manage pesticides and favors consideration of *P. acuta* as a suitable organism to perform ecotoxicological assays, given its role as an essential organism in freshwater ecosystem food webs.

## Material and Methods

### Chemicals and reagents

A 250 mg/ml endosulfan solution (Cat N° 32015, Fluka Analytical) was prepared in acetone. This highly concentrated solution was used to prepare the three acetone stock solutions: 10 µg/mL, 100 µg/mL, and 1 mg/mL. Exposure solutions were prepared by 1:1,000 dilution of these stocks in the artificial pond water (see below).

TRIzol and M-MLV enzyme were obtained from Invitrogen (Germany), oligonucleotide dT18 primer and gene-specific primers were supplied by Sigma Genosys (UK), RNase-free DNase was purchased from Sigma, DNA polymerase and dNTPs were obtained from Biotools (Spain), and EvaGreen was purchased from Biotium (USA).

### Animals

Adult *P. acuta*, a pulmonata freshwater snail, was used for this study. The populations were established from animals provided by Dr. Sánchez-Argüello (Instituto Nacional de Investigación y Tecnología Agraria y Alimentaria, Spain) and were maintained in the laboratory of Biology and Environmental Toxicology under constant conditions for numerous generations. *Physella acuta* is a hermaphroditic species, and the gelatinous egg masses that they laid were collected and allocated in a 6 L aquarium previously filled with 2 L of artificial pond water (2 mM CaCl_2_, 0.5 mM MgSO_4_, 0.77 mM NaHCO_3_, and 0.08 mM KCl). They were maintained under constant aeration at 19 ± 1 °C under a 16 h light and 8 h dark cycle. The feeding regime was described previously^62^.

### Treatments

Approximately 0.7 cm of length and two-month-old adults were used (since the organisms enter reproductive maturity at this time). Three experiments were carried out. Each experiment was performed with six snails per treatment using glass vessels that contained 200 mL artificial pond water. The number of animals analyzed by concentration was nine. The animals were exposed to the pesticide for 7 days, during which time they were fed every 2 days, and the media and endosulfan were changed every 48 h. The concentrations selected for this experiment were 1, 10, and 100 μg/L (1, 10 and 100 ppb or 2.4, 24.6 and 246.3 nM, respectively). These concentrations were selected based on previous values assayed for the freshwater snail (between 0.4 and 0.8 mg/L^63^), considering that lower concentrations are closer to environmental conditions. Artificial pond water with the same amount of solvent was used as the control. Three experiments were performed with individuals from three different breeding batches. There was no mortality for any treatments during the experiments. The whole animals (without shell) were frozen using dry ice, homogenized in 300 μL TRIzol, and stored at −80 °C until RNA was isolated.

### Identification of new genes

The *GSTK1*, *GSTM1*, *HSP60*, and *GRP78* sequences were obtained from the same transcriptome and following the same procedure as described in Aquilino *et al*., 2019^22^. In brief, RNA was extracted after pooling individuals from control, Cadmium (Cd), Bisphenol A (BPA), or Triclosan (TCS) treatments: one for a mixture of egg masses, one for a mixture of juveniles, and two for a mixture of adults. The exposures were set for 24 hours for Cd (1 μM), BPA (10 μg/L), and TCS (10 μg/L). The RNA was sent to BaseClear (www.baseclear.com), and sequenced on an Illumina Hi-Seq. 2500 using a 125 cycle paired ended protocol. The de novo transcriptome was assembled using Trinity version r2014-04-13^64^ under default settings. All reads generated for this study were deposited in the European Nucleotide Archive (ENA) within study PRJEB23140. The sequences were deposited in GenBank with the accession numbers MK814856 (*Glutathione – S – transferase kappa 1*), MK814857 (*Glutathione – S – transferase mu 1*), MK814858 (*Heat shock protein 60*), and MK814859 (*Glucose-regulated protein 78/Binding immunoglobulin protein*).

### RNA isolation and complementary DNA (cDNA) synthesis

Each homogenate snail in TRIzol was used to isolate RNA following the manufacturer’s instructions. Briefly, 0.2 volume of chloroform were added to the 300 μL homogenate and thoroughly mixed, incubated for 2–3 min at room temperature, and centrifuged at 10,000 rpm at 4 °C. The aqueous upper phase was recovered, and RNA was precipitated with isopropanol and washed with 70% ethanol. DNAse treatment was performed using RNase-free DNase (Roche, Germany), followed by a phenol/chloroform/isoamyl extraction with Phase Lock Light tubes (5prime, Spain). The isolated RNA was resuspended in 25 μL diethyl pyrocarbonate (DEPC)-treated water, quantified by UV spectrometry (Biphotometer, Eppendorf) and stored at −80 °C.

RNA was used to synthesize cDNA. The reaction was performed in a 40 µL volume with 8 µg RNA, 2 µL oligonucleotide dT18 primer (0.5 µg/µL), 200 units M-MLV enzyme, and 2 µL 10 mM dNTPs. The reaction was incubated at 37 °C for 50 min and stopped at 75 °C for 15 min. cDNA was stored at –20 °C until use in RT-PCR.

### RT-PCR

The cDNA (0.6 µL/well) was used as a template for RT-PCR. The reaction was performed with a CFX96 thermocycler (Bio-Rad, USA) by using 0.5 unit DNA polymerase, 0.4 mM dNTPs, and 0.5X EvaGreen. The thermal cycling program included an initial denaturation at 95 °C for 30 s followed by 40 cycles of 95 °C denaturation for 15 s, 50 °C annealing for 15 s, and 72 °C elongation for 30 s. Finally, a melting curve was generated to confirm the presence of a single peak. Reference genes were glyceraldehyde-3-phosphate dehydrogenase (*GAPDH*) and ribosomal protein L10 (*rpL10*). For *GSTk1*, *GSTm1*, *HSP60*, and *GRP78*, efficiency was calculated by using five different 1:2 dilutions by duplicate. Each sample was run in duplicate wells, and two independent replicates were used for each experiment. Primers and efficiencies are listed in Table 2. Bio-Rad CFX Maestro software was used to analyze and determine total mRNA levels of normalized gene expression (2^−ΔΔCq^).

**Table 2:** Primer sequences and PCR efficiency for the genes used in this study.

Primer	Primer sequence	Efficiency (%)	Reference
rpL10 F rpL10 R	TGCACGTGAGGCTGATGAAA GTGGCCACTTTGTGAAACCC	102.3	Aquilino *et al*., 2019^22^
GAPDH F GAPDH R	ATACATCAGGAACAGGGACTC GACTTATGACAACCGTGCA	93.9	
Hsp16.6 F Hsp16.6 R	GCATGAGGAGAAGCAAGACA CAGTACACCATGGGCATTCA	96.4	
MRP1 F MRP1 R	CAGGGGCAGGTAAGTCATCC AGTGAGCCTTGATCGCACAT	94.5	
Hsp90 F Hsp90 R	GTTTGTGTCACTAAAGAAGGCC TGTCACTAGCCTATTTGATACAACC	91.8	
cyp2U1 F cyp2U1 R	GTGCATCCTCTACGCGATCA GGCTAGTTTGGGCCTGTCTT	102.1	
cyp3a7 F cyp3a7 R	ACGGCTTGGCCTCTCAATAC CGGTTTCTTTCTCGGCGTTC	84.8	
cyp4f22 F cyp4f22 R	AGCAGAAAAAGCTCAGCCCT CTTGGTTTTGGCAGCCAGTC	87.2	
**Primer**	**Primer sequence**	**Efficiency (%)**	
GSTk1 F GSTk1 R	TGAGCAGAGTAGTTTGGCTGC	96.7	
	ATGCCCCTAATTCTGTGGCT		
GSTm1 F GSTm1 R	ATTGGGCCATTAGAGGGCTT	93.1	
	GTTGGACCATCTCCTTGCAC		
Hsp60 F Hsp60 R	ATTGCTTATCGTGGCTGAGG	82	
	TGGCAATAGCCATATCCTGC		
Grp78 F Grp78 R	TGGTGGCTCAACCCGTATTC	96.8	
	CCCCACTCAAAACACCAGCT		

### Statistical analysis

Statistical analysis was done using SPSS 24 (IBM, USA). Shapiro-Wilk and Levene tests were performed to test whether the data was normally distributed and to obtain the variance homogeneity. For normally distributed data, significant differences were determined using analysis of variance (ANOVA), and in accordance with the Levene test, either the Bonferroni or Games-Howell modification was used. The non-normally distributed data were analyzed with the nonparametric Kruskal-Wallis test. Statistical significance was set at p ≤ 0.05.
